# A Few Words Introductory

**Published:** 1874-03

**Authors:** Swan M. Burnett


### A FEW WORDS INTRODUCTORY.

This number of the RECORD appears under the new regime, mention of which was made in the last issue.

The managing editors, Drs. Powell & Goldsmith, anxious to serve their subscribers and the profession in the best possible manner, have associated with them in the editorial department of their Journal, several medical gentlemen, who will represent the interest of the RECORD in different States and sections of the country.

By this means a community of interest and breadth and liberality of opinion is given to the Journal, that it would by no means have under an individual management, or the management of any number of gentlemen in the same locality. It is thus placed beyond the possibility of being the organ of any clique or ring, and is made to represent interests so wide and diversified that its character ceases to be sectional, even, and becomes truly professional, and thoroughly and essentially national.

A Journal of such a character is a want the profession has long felt in the South, and such an one it is the design of the editors of this periodical to furnish. No Medical Journal can flourish which represents primarily the interests of any combination, whether of a section, a college, or a society or any number of private individuals. What the profession demands—and of right—is a Journal that is devoted singly and exclusively to their interests as a whole. They want the latest views of the great minds of the profession, respecting the general principles upon which the science of medicine is based; for the most recent and reliable researches into the hidden mysteries of anatomy, physiology and pathology; for the latest observations regarding the therapia of diseases; and for the light which the host of collateral science throws upon all questions connected with the healing art. To merit their respect and confidence, and gain their support, a Journal must furnish these, and unless it does, it cannot in reason expect to deserve the one or obtain the other.

As editors, we feel and acknowledge these to be facts, and acting upon this belief, and desiring above all things to make a Journal that shall be thoroughly acceptable to the profession, we shall use our utmost endeavors to come up to these demands set upon us by the calling which we feel it our highest duty and greatest pleasure to honor. But to come up to the standard we have set, will require the hearty and earnest co-operation of our brethren in the profession everywhere. "Brick *can not* be made without straw," neither can a Medical Journal be made what it should be without the help and assistance of its friends. We want our frends, not our friends simply, but the friends of progressive medicine everywhere, to aid us in building up a work in the South, of which the profession at large may be proud. Write for us articles embodying the facts of your observation and experience, and subscribe for the RECORD, and obtain the subscription of your friends. Take a real live interest in your Medical Journal, and you may rest assured it will take a live interest in you. With these remarks, by way of an introductory, we wish one and all the compliments of the season, and hope our associations may be as pleasant during the coming year as they have been in the one that has just past.

SWAN M. BURNETT.

The above was intended for the January number, but owing to its having until a few days ago, failed to reach us, is published in the present issue.—LOCAL EDS.

